# Wherein Does Dentistry Differ from Trade?

**Published:** 1901-11

**Authors:** 


### Editorial.

### WHEREIN DOES DENTISTRY DIFFER FROM TRADE?

In a recent article from the pen of one of the prominent members of the dental profession [1] the question was asked, "What is a profession?" The answer was given, "It is an aggregation of men who have been specially educated to do special work,—nothing more and nothing less." If this definition be correct it conflicts with the general idea of a profession which is clearly outlined by the Century Dictionary, as follows: "In professions, strictly so-called, a preliminary examination as to qualification is usually demanded by law or usage, and a license or other official authority founded thereon required." Worcester's Dictionary defines a profession to be "a calling; a vocation; an occupation; a business; office; employment,—especially an employment requiring a learned education, as those of divinity, law, and physics."

Our respected colleague, in his able review of dentistry as a profession and its connection with trade, evidently overlooked the wide distinction made by the Century that examinations, preliminary and final, are requisites as part of the process required to establish a professional vocation. While this may not be satisfactory to many, and may seem an altogether arbitrary distinction between classes, it does nevertheless mark a dividing line between all callings that require special scientific training and examinations to de-

---

[1] Safford G. Perry, D.D.S., New York.

termine the result and those departments of industry that demand years of practical training without the usual requirements of the professional schools.

The difference between the mechanic, tradesman, banker, etc., and the lawyer, doctor, civil, mechanical, and electrical engineers may not be distinctly drawn by the average mind, yet the difference is recognized; but this fact by no means rightfully establishes any claim to superiority of one class over the other.

There seems a natural tendency with some minds to regard all class distinctions as aristocratic in character and tendency, and are, therefore, not to be tolerated in this country. This view is. in the larger sense, true of the world as a whole, but these social separations as understood in monarchical countries have no place, and should have no place, in a republic; and yet there are spheres of mental environments that cannot be overstepped, even should the high and low respectively desire the change.

The writer can have no sympathy with the affectation of superiority assumed by some college-trained men over the practical man, the "mere mechanic." It is not a question of superiority in any degree. If this were so, then the positions would frequently have to be reversed, for the practical man, the man who creates, rises immeasurably superior, in the world's work to the man learned as the school-men. It is an unwise effort to attempt to create class distinctions in the great and multiplying work of civilization. The humblest worker has his place as well as the highest, all being parts of a complex whole, of which nothing can be lost without becoming a serious set-back to the world's progress. Following these prefatory thoughts, the question may properly be asked, What relation does dentistry hold to the trades closely connected with it? The essayist quoted answers this by saying, "The real truth is that we are linked indissolubly together, and neither one could get on without the other." This is a recognized truth as dentistry is organized today, but the writer forgets that there was a period within the memory of many now living when a supply house was an unknown quantity, and yet dentistry flourished. The fact is that the supply houses have simply become the assembling houses of dental ideas. It is a fallacy to assume that if all the dental supply houses were to be wiped from the face of the earth that thereby the destruction of dentistry would be assured. The modern dentist doubtless feels that this would be the result, but if he will stop a moment to consider, he will find that the question of demand and supply always regulates itself. In the decades prior to 1830 there were no supply houses, yet instruments were procured, gold was prepared, chairs were made; in fact, all the petty details, now regarded so important, were individually attended to and were satisfactory for the time and conditions.

What the supply houses have done has been to broaden out this individual work, bringing to bear the highest mechanical excellence, specializing so that in assembling the results the highest skill attainable has been reached, thus enabling the practitioner to do better and more effectual work than could possibly have been done under the methods common to the period named.

While this is cordially recognized by the writer, he must constantly bear in mind the fact that the supply houses have been dependent upon dentistry for almost every advance made in this special work. For confirmation of this statement it is only necessary to go back to the origin of these houses. In this country the first efforts were principally confined to the manufacture and sale of porcelain teeth. The dentists of France and the earlier dentists of this country originated and perfected the processes necessary to make an artificial tooth, and to Dr. Wildman of Philadelphia belongs the honor of so perfecting tooth body and tooth gum that no advance has been made since his day. All changes of any value in instruments, from the original forceps to the most valuable operative instruments, have been devised or improved by dentists. While it is true, as our essayist remarks, "The world has never witnessed such a spectacle as the growth of the enormous trade interests that have sprung into existence to satisfy our demands," it is equally true that this growth has been entirely dependent upon the development of the profession of dentistry outside and apart from any supply houses. The demand came from the profession, and it was simply the part of good business to meet it with the best attainable. It is worthy of all commendation that this effort has been remarkably successful, and the dentist of to-day would not willingly return to the original conditions when these supply houses were not.

The distinction has not been properly made between dentistry and its commercial connections. The average dentist is apparently lost in a maze of ethical contradictions whenever this subject is broached. Our essayist seems to have been in a mental confusion regarding it, and practically came to the conclusion that there was no such thing as a profession, and that we, as dentists, were stultifying ourselves that a difference existed. The true position to assume, as the writer views it, is not one of superiority, but one in which the dividing line is distinctly drawn and cannot be overstepped by either party without injury. The man of trade has his methods, which are not compatible with the work of the profession, and yet, properly conducted, these are strictly worthy of high honor. On the other hand, the methods of the professional man would be destructive to trade interests if adopted by business houses. Both are honest, both are living up to their highest ideals, and both are right within the limits of their environments. It is evident, from what has been said, that the two divisions are dependent upon each other, but neither can absorb the other or directly share in the results.

The illustrations are many of similar conditions in other walks of professional and commercial life. The surgeon is entirely dependent on the instrument-maker for his tools, and were this supply to fail him he would be incapable of continuing his work. The general practitioner of medicine would find it very embarrassing were the pharmaceutical establishment not in existence. To the doctor the druggist is the man of supplies. The ophthalmologist would write in vain his prescriptions were the optical establishment not in existence; and yet the surgical-instrument-maker's announcement would not be accepted by the surgeon as representing his profession. The druggist's circular or periodical would have a value to the man of that profession, but it would in nowise represent the medical practitioner's work; and equally so would be the optical journal in its relation to ophthalmology. The lines are strictly and naturally drawn between all these honorable callings, and it is simply a mental delusion to suppose that things not directly related can be moved out of their natural order.

In view of these facts, it is strange that there should be a constant effort made to enforce upon the dental mind the unsupported theory that there is no difference between commercialism and the profession. This has been carried so far that the large majority of dentists fail to make any distinction between the literature that originates in a supply house and that which comes directly through the medium of professional effort. The stream cannot rise higher than its source, and the journal published by a supply house cannot represent anything but trade. It is an incongruous combination of words to call such a journal a representative of the dental profession. Dentistry has been misled too long by appearances. The dental journals representing the supply houses have unquestionably held an important place in dental progress. Several of these are ably edited, and are naturally regarded by the majority as the leaders of professional thought in this country. They openly claim to be published in the interest of the dental profession, and are devoted to its interests. This can never be more than a partial truth, and partial truths involve a moral loss not to be compensated for by literary ability or interesting matter.

It is possible the time may come when dentistry will be submerged theoretically into the supply houses and completely so far as its periodical literature is concerned. The outlook for a better state of things is not promising. Money is a recognized force in subverting the ethical sense, and so long as this remains a power the dentistry of the twentieth century will fail to accomplish its highest and most ennobling work.

There is no reason for antagonisms, but, on the contrary, mutual regard and co-operation should be extended; but it should never be forgotten that there is a limit to this, and neither the supply houses nor dentists can afford to overstep the fixed professional boundary line without loss. The actions of individuals and dental societies in thus violating professional ethics has resulted in demoralization in our ranks. It is time for a careful introspection individually and collectively to endeavor, if possible, to discover our true paths of duty to the dental profession, to which we all owe undivided allegiance.

It is very evident that a higher standard of professional life is needed, and if dentistry is ever to reach the true status it will not be through the kindly motives, but mistaken aims, of those who try to harmonize the inharmonious and to bring those together who are separated by a positive and indestructible law.

